# Improving Patient Understanding of Emergency Department Discharge Instructions

**DOI:** 10.5811/westjem.18579

**Published:** 2024-09-24

**Authors:** Sarah Russell, Nancy Jacobson, Ashley Pavlic

**Affiliations:** Medical College of Wisconsin, Department of Emergency Medicine, Milwaukee, Wisconsin

## Abstract

**Introduction:**

Previous studies have shown that patients in the emergency department (ED) are frequently given incomplete discharge instructions that are written at least four grade levels above the recommended sixth-grade reading level, leading to poor understanding. Our aims in this study were to implement standardized discharge instructions containing six key components written at a more appropriate reading level for common emergency department (ED) diagnoses to improve patient understanding.

**Methods:**

We conducted this study in a 20-bed ED at an urban Veteran’s Administration hospital. Data was collected via in-person patient and clinician interviews. Patient interviews were conducted after patients received their discharge instructions. We compared patient responses to clinician responses and marked them as incorrect, partially correct, or correct with a score of 0, 0.5, or 1, respectively. The maximum possible score for each interview was six. Six key components of discharge instructions were asked about: diagnosis; new medications; at-home care; duration of illness; reasons to return; and follow-up. There were 25 patients in the pre-intervention group and 20 in the intervention group with the standardized set of instructions. We performed a Mann-Whitney U test on the total interview scores in the control and intervention groups and conducted a sub-analysis on the individual scores for each of the six key components.

**Results:**

The patients in the intervention demonstrated a statistically significant increase in patient-clinician correlation when compared to the patients in the pre-intervention group overall (*P* < 0.05), and two of the six key components of the discharge instructions individually showed statistically significant increase in patient-clinician correlation when standardized discharge instructions were used.

**Conclusion:**

Patients who received the standardized discharge instructions had improved understanding of their discharge instructions. Future opportunities extending off this pilot study include expanding the number of diagnoses for which standardized instructions are used and investigating patient-centered outcomes related to these instructions.

Population Health Research Capsule What do we already know about this issue?

*Patient understanding of ED discharge instructions is important for patient care, outcomes, and experience.*

What was the research question?

*Does implementing standardized discharge instruction templates improve patient understanding at time of discharge?*

What was the major finding of the study?

*The intervention group demonstrated a statistically significant increase in understanding of their instructions (*P* < 0.05).*

How does this improve population health?

*Good understanding of ED discharge instructions is vital to patient health and empowers patients by allowing them to better understand their disease and its course.*

## INTRODUCTION

Several studies have analyzed the effectiveness of discharge instructions given to emergency department (ED) patients at the time of discharge and have identified areas for improvement.^1^ These studies recommend that key components of discharge instructions include diagnosis, expected duration of illness, at-home care, return precautions, and follow-up plan. Nonetheless, many ED patients do not receive discharge instructions that include all these components.^2^ ^,^ ^3^ In addition to being incomplete, discharge instructions are often difficult to read.^4^ ^,^ ^5^ In fact, discharge information given to trauma patients at one institution was written at least four grade levels higher on average than the National Institutes of Health-recommended sixth grade reading level. They noted that after improving readability by breaking up complex sentences, using simple words, and using bullet points and subheadings, there was a significant decrease in post-discharge return phone calls and readmissions.^5^ Additionally, having a good understanding of one’s discharge instructions can help promote optimal health and recovery following an ED visit. Patients may also have fewer unnecessary return visits to the ED if they better understand their discharge instructions.^6^

Currently, discharge instructions at this urban Veteran’s Administration (VA) hospital include a section at the beginning of the instructions where clinicians can free text any specific instructions they have for the patient. This section may also be kept blank. There is also standardized information about the discharge diagnosis, which is included in all instructions. In this pilot study we aimed to determine whether implementing discharge instructions that are standardized at an appropriate reading level and include key components would improve patient understanding of discharge instructions (measured by patient-clinician correlation).

## METHODS

We conducted this pilot study at a 20-bed ED urban VA hospital. This study did not collect any personal patient data and thus was deemed by the VA internal review board office to be institutional review board- exempt. Study participants were approached by nursing staff, clinicians, or study staff and asked whether they would be willing to participate in a short interview to help a quality improvement project focused on discharge instructions. If the patient agreed, they were interviewed by study staff regarding the key components of discharge instructions. They were asked to state their diagnosis, what (if any) new medications were prescribed, what they needed to do at home to take care of their illness, expected duration of illness, reasons to return to the ED, and who to follow up with. Study staff recorded their answers. Patients were permitted to look at their discharge instructions at any time during the interview to help answer the questions and were reminded of this at the start of the interview. Study staff then asked the clinician (physician or advanced practice practioner [APP]) the same questions.

For the initial control group, clinicians were free to include whatever they wanted in the free-text portion of the discharge instructions. This group of 25 patients had the following discharge diagnoses: edema; motor vehicle collision; concussion; strain; acute psychosis; constipation; fracture; shingles; hyperglycemia; cystic acne; cervical radiculopathy; oral mucosal lesions; conjunctivitis; sinusitis; pneumonia; ear infection; cellulitis; fatigue; diarrhea; chest pain; back pain; balanoposthitis; chronic obstructive pulmonary disease (COPD); and dehydration. The clinicians treating this group included 10 physicians and two APPs. Data was again collected by study staff (Russell) in the form of in-person interviews and addressed the six key components.

A set of standardized discharge instructions were developed for 12 common ED diagnoses and edited to contain six key components. These templates were created with subheadings and bullet points to make the instructions easier to follow and understand (Appendix A). The discharge diagnoses addressed in this group included many of the most common emergency department diagnoses: abdominal pain; back pain; cellulitis; chest pain; congestive heart failure; COPD; concussion; fracture; headache; no fracture (sprain/strain); rib fracture; and vertigo. These discharge instruction templates were reviewed for accuracy and completeness by three board-certified emergency physicians, including one study staff, one director of ED operations, and one educational director.

A convenience sample of emergency clinicians, including both board-certified physicians and physician assistants, voluntarily participated in the post-standardized intervention phase. Volunteer clinicians had the standardized discharge instructions uploaded into their dictation software Dragon (Nuance Communications, Inc, Burlington, MA) and used these standardized instructions when study staff was on site to conduct interviews. The study staff then collected data via in-person interviews for these clinicians and for the 20 patients for whom the standardized discharge instructions were used.

In both groups, patient responses were compared to their own clinician’s responses and marked and coded as incorrect (0), partially correct (0.5), or correct (1) with a maximum total score of six. Results were scored by each member of the study team independently as well as by a third, board-certified emergency physicians who was the director of ED operations. We performed a Mann-Whitney U test on the total interview scores in the control and intervention groups and conducted a sub-analysis on the individual scores for each of the six key components.

## RESULTS

Demographics: The treatment clinicians for the patients in the baseline group included 10 physicians and two APPs. The treatment clinicians in the post-standardized intervention group included three physicians and two APPs. Note that some clinicians were involved in both groups.

Patients in the pre-standardization group already showed high levels of understanding in three areas (above .75): their diagnosis; new medications; and who to follow up with. The patients in the post-standardized group overall demonstrated a statistically significant increase in patient-clinician concordance when compared to the patients in the baseline group (*P* < 0.05) (Figure), and two of the three low understanding areas— duration of illness and reasons to return—had statistically significant increases in patient-clinician concordance in the baseline vs post-standardized group.

**Figure.**
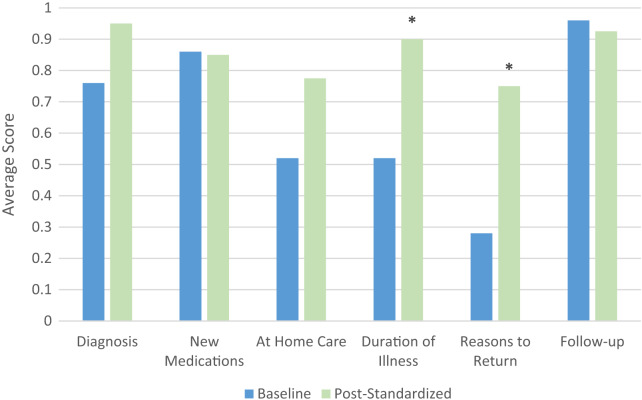
Average score by question (*signifies statistical significance).

## DISCUSSION

The data from this pilot study suggests that implementing discharge instructions standardized to increase readability and include key components improved patient understanding compared to discharge instructions entered in via free text by the clinician. Like other studies, our study demonstrated that reasons to return were among the most poorly understood.^7^ As seen in the Figure, there is clear improvement in this area with the implementation of standardized instructions. This is essential to patient care in the ED. Transitions of care have been identified as critically important times for transfer of information.^8^ This is especially true when patients are transitioning from hospital-based care in the ED to home. Indeed, patient understanding of discharge instructions has been shown to improve health outcomes including minimizing return visits, increasing follow-up, and enabling improved at-home compliance with their clinician’s plan of care.^6^

Further, institutions such as the Centers for Medicare and Medicaid Services have identified patient understanding of discharge instructions as a key domain of patient experience, and patients are asked how well they were able to understand the discharge instructions provided during their ED visit on the ED Consumer Assessment of Healthcare Providers and Systems Survey. One recent study implemented a mnemonic “DC HOME” (discharge diagnosis, care rendered, health and lifestyle modifications, obstacles after discharge, prescribed medications, and expectations) and formalized education regarding its implementation among resident physicians, which demonstrated success in both inclusion of these components and patient satisfaction.^9^ This intervention included several of the components we included in our standardized written instructions.

Having a good understanding of one’s discharge instructions is important for many reasons, including that patients can have optimal health and recovery following their ED visit. Better understanding of discharge instructions can also decrease unnecessary return visits to the ED by empowering patients with the information they need to make appropriate follow-up appointments and to better understand the expected course of their illness, which may decrease the unnecessary cost of an additional ED visit for the patient.

## LIMITATIONS

One limitation of this study is that inter-rater reliability was not assessed within the data collection and statistical analysis. We did not collect this data and, therefore, it is unclear how closely the doctors’ ratings correlated to one another. Future analysis and interventions would benefit from two doctors rating the understanding and then performing kappa statistics to measure the level of agreement between the two doctors. An additional limitation of this study is its small sample sizes. We used small sample sizes as this was a pilot study with the goal of assessing significant impact as well as feasibility of implementation. As this pilot demonstrates statistical significance and clear beneficial impact to patient understanding, we now have a foundation for future expansion and additional research within this area.

Based on this pilot study we recognize several future opportunities. While this study was focused on standardizing 12 common discharge diagnoses, a future work could expand the number of diagnoses as well as the number of clinicians. There is an opportunity to examine patient-centered outcomes including following patients after discharge to assess knowledge retention, return ED visits, and adherence with recommended follow-up. This pilot study demonstrates a first step in better understanding these patient centered outcomes potentially impacted by discharge instructions. Further, nursing staff were the primary individuals distributing the written discharge instructions to the patients and explaining them one final time prior to discharge. There is currently widely variable practice on how nursing staff provide and discuss these instructions with the patients. This study did not address this variability as our goal was to evaluate how changing the single variable of the written discharge instructions would affect patient understanding. Future work may include standardizing how clinicians or nursing staff provide discharge instructions as this has also been shown to impact patient understanding and satisfaction.^9^

## CONCLUSION

Overall, this crucial pilot study suggests that standardized discharge instructions significantly improve patients’ understanding of their instructions overall and, specifically, the expected duration of illness and reasons to return. This intervention is easy to implement, cost effective, empowers patients to better understand their health condition, impacts core ED quality measures, and should be further studied.

## Supplementary Information




