# Supplementary material for: Improving Patient Understanding of Emergency Department Discharge Instructions
Source: West J Emerg Med. 2024 Sep 24;25(6):917–20. doi: 10.5811/westjem.18579 (PMC11610731; doi:10.5811/westjem.18579)
Supplement: Supplementary file 1 [file wjem-25-917-s001.docx]

**Appendix A.**

Pre-Existing Template:

Your Diagnosis is: Back pain.

**Return to the Emergency Department for** fever, unable to walk or weakness in your arms or legs, new changes in sensation, loss of the function of your bladder or bowels, worsened or severe pain that is not controlled by home medications, if symptoms worsen or for any other concerns.

**Medications**:

These are your new prescriptions: ***

These medicines that you take now have been changed: ***

Please refer to the medication section for instructions on how to take them.

**Major procedures performed during your ED visit:** ***

**Additional instructions**:

Call your primary doctor to set up an appt for follow up of your back pain. If back pain has been an on going problem for you, you may benefit from receiving a referral to a pain management specialist. Call your primary doctor for any more pain medication refills.

Only take ibuprofen or naproxen, don't take both at the same time because these are the same medications. You may take 1 naproxen every 12 hours OR 600mg Ibuprofen every 6 hours or 800mg ibuprofen every 8 hours as needed for pain. You should also take tylenol 1000mg every 6 hours as needed for pain.

You may do gentle stretching which could help relieve the pain. You have no activity restrictions. You can move around as much or as little as you want, please use pain as your guide. If it hurts too much, don't do it. You may also use heat to relieve your pain. Exercises like yoga may also help your pain.

Standardized Template

**Your diagnosis is:** Back pain

**Expected duration of illness:** Back pain often lasts for days to weeks. Please follow up closely with your primary doctor.

**At-home instructions:**

1. For pain relief you may take 600 mg of ibuprofen (with food) every 6 hours OR 1 naproxen every 12 hours (with food). This will help decrease any inflammation. In addition, you may also take acetaminophen 1000 mg every 6 hours as needed for pain (be sure to take no more than 4,000 mg of acetaminophen in a 24 hr period.
2. You have no activity restrictions. However, if a certain movement hurts too much don’t do it. You may do gentle stretching which could help relieve the pain.
3. You may also use a heating pad or ice on the affected area, which ever feels better. Apply it for 15-20 minutes 3-4 times per day.
4. If you are over-weight, the best thing you can do for your back will be to lose weight.

****Return to the Emergency Department for:** fever, if you are unable to walk or develop weakness in your arms or legs, new changes in sensation, cannot control your bladder or bowels, or for worsening or severe pain that is not made better by your home medications.
